# The Spatial Landscape of the Bacterial Community and Bile Acids in the Digestive Tract of Patients With Bile Reflux

**DOI:** 10.3389/fmicb.2022.835310

**Published:** 2022-03-09

**Authors:** Ni Yang, Jun Xu, Xuemei Wang, Ning Chen, Lin Su, Yulan Liu

**Affiliations:** ^1^Department of Gastroenterology, Peking University People’s Hospital, Beijing, China; ^2^Clinical Center of Immune-Mediated Digestive Diseases, Peking University People’s Hospital, Beijing, China

**Keywords:** bile reflux, microbiota, bile acid, 16S rRNA sequencing, bacteria

## Abstract

**Background:**

Bile reflux can lead to inflammation and increased intestinal metaplasia. Since bile acids can influence the gastrointestinal environment, it is possible that bile reflux may alter the gastric microbiota and potentially the oral or gut microbiota. Bile acids have a very complex interrelationship with microbiota. We aimed to explore the characteristics of the digestive tract microbiota and bile acids profile in bile reflux patients.

**Methods:**

This study included 20 chronic gastritis patients with bile reflux and 20 chronic gastritis patients without bile reflux. Saliva, gastric fluid, and fecal samples were collected for bile acid testing. Buccal mucosal swabs, gastric mucosal tissues, and feces were collected for bacteria detection. The UPLC-MS/MS examined bile acids profiles. 16S rRNA gene sequencing was used to analyze the bacterial profile.

**Results:**

Bilirubin in the blood increased in bile reflux patients. No other clinical factors were identified to be significantly associated with bile reflux. 12-DHCA, 6,7-diketo LCA, and βHDCA decreased while TUDCA increased in saliva of bile reflux patients. *Streptococcus*, *Capnocytophaga*, *Neisseria*, and *Actinobacillus* decreased in oral mucosa of bile reflux patients while *Helicobacter*, *Prevotella*, and *Veillonella* increased. Gastric bile acid levels were generally higher in bile reflux patients. Gastric mucosal microbiota was highly stable. The changes in fecal bile acids were insignificant. *Bifidobacterium*, *Prevotella_2*, *Ruminococcus*, *Weissella*, *Neisseria*, and *Akkermansia* decreased in fecal samples from bile reflux patients; while *Alloprevotella*, *Prevotella_9*, *Parabacteroides*, and *Megamonas* increased.

**Conclusion:**

Our results demonstrate that bile reflux significantly alters the oral, gastric, and intestinal bile acids profiles but only influences the oral and gut microbiota composition. These findings indicate that bile reflux can modulate the gastrointestinal microbiota in a site-specific manner.

## Introduction

Bile reflux is the reflux of duodenal fluid containing excess bile into the stomach. Gastrointestinal movement dysfunction and gastrointestinal hormone secretion may contribute to bile reflux (Dewar et al., 1983; Wilson et al., 1993). Bile reflux gastritis induced by partial gastrectomy is called secondary bile reflux gastritis. After bile acids go into the stomach, they turn into free bile acids in an acidic environment, destroying the structure of epithelial cells. Bile acid conjugation with gastric acid enhances the viability of acid hydrolase and destroys lysosomal membrane, resulting in hydrogen ion diffusion in the reverse direction. Activated mast cells release histamine and cause gastric acid and pepsin secretion, which aggravate mucosal damage. The diagnosis of bile reflux lacks internationally acknowledged standards. Nowadays, gastroscopy, Bilitec 2000, and radionuclide scanning are used for diagnosis (Madura, 2003).

Bile reflux damages gastric mucosa and causes chemical gastritis. Histologically, it can be manifested as gastric fovea hyperplasia, gastric mucosal edema, lamina propria muscle fiber hyperplasia, vasodilation, and hyperemia but sometimes inflammatory cell infiltration is not obvious. Bile reflux is considered to be related to intestinal metaplasia, which might be a precancerous lesion. A multicenter study observed endoscopic biopsy pathology in 2,283 patients and found that the proportion of intestinal metaplasia increased in patients with severe bile reflux regardless of *Helicobacter pylori* (Hp) infection (Matsuhisa et al., 2013).

Bile reflux also stimulates antral G cells’ gastrin secretion, promoting gastric acid secretion and inhibiting pyloric sphincter contraction. A low acid environment and the reflux of intestinal bacteria result in excessive stomach growth (Carboni et al., 1986). Valerie et al. (Poxon et al., 1986) collected gastric contents from patients undergoing antrectomy for duodenal ulcers and healthy volunteers. They found that the total bacterial count in patients significantly increased, especially *Streptococcus faecalis* and *Veillonella*, which deconjugate and hydrolyze bile acids. The total bile acid concentration in gastric juice also increased with easier detection of free secondary bile acid. There are complex relationships between bile acids and microbiota. Bile reflux may also affect intestinal or oral microecology and bile acids profile. Therefore, we intend to explore the bacteria and bile acid profile of digestive tract in patients with bile reflux, trying to clarify the clinical significance of the alteration.

## Materials and Methods

### Study Design and Sample Collection

Patients were enrolled in Peking University People’s Hospital between September 2019 and October 2020 who undergo painless gastroscopy. They were asked to avoid using acid inhibitors, antibiotics, and probiotics within 4 weeks. Subjects were enrolled with intact clinical information, as well as saliva and buccal mucosa swab samples, and then were divided into two groups according to endoscopic findings. There was bile staining in the bile reflux (REF) group in the gastric juice or gastric mucosa (Kellosalo et al., 1991). The controls (CON) were the patients who were diagnosed with chronic gastritis with clear gastric juice. The gastric juice and gastric mucosa of patients were collected under gastroscopy, and the fecal samples were collected after the examination. The bile reflux patients were 1:1 matched with controls according to gender and age (±5). The sample size was evaluated based on previous reasearch (Zhao et al., 2020). Considering the 10% loss of follow-up rate, therefore, number was equal to 20 in each group. This study was approved by the Ethics Committee of Peking University People’s hospital (Document ID: 2019PHB049-02).

### Information Collecting

The same questionnaire was used to collect basic information, clinical symptoms, psychological conditions, and patients’ living habits. Psychological assessment was performed using PHQ-2 and GAD-2 questionnaires (Kroenke et al., 2003; Donker et al., 2011). Blood biochemical indexes of patients were also collected.

### Bile Acids Profiling

Bile acid profiles were examined using an ultra-performance liquid chromatography-tandem mass spectrometer (UPLC-MS/MS, acquity UPLC Xevo tq-s, waters Corp., Milford, Ma, United States). Raw data files generated by UPLC-MS/MS were processed in Masslynx software (v4.1, Waters, Milford, MA, United States), which integrated and quantified each bile acid. The actual concentrations were obtained by comparing the metabolites in samples of unknown concentrations with a set of standard samples of known concentrations (quantification curve).

### 16S rRNA Sequencing and Bioinformatic Analysis

The total microbial genomic DNA was extracted from stool samples using the PSP Spin Stool DNA Kit (German, Stratecolecular). The extracted bacterial DNA was analyzed by Illumina high-throughput sequencing based on the V3 and V4 regions of 16S rRNA (357F and 806R). Each PCR product was purified and amplified again to link with sample-specific barcodes. Sequencing was performed using the instrument secondary analysis of MiSeq Reporter software (MSR). Software for bacterial sequence analysis was vsearch v2.8.1 and usearch v10 (bit 32; Edgar, 2013). Exact sequence variants method was performed to filter chimeras (Edgar, 2016). Amplicon sequence variants (ASVs) were aligened with database of rdp_16s_v16_sp.fa. The detail process and data were shown in Supplementary Table 2. Based on the ASVs tables, the beta diversity with PCoA was calculated with Bray Curtis distance and tested with PERMANONVA (Adonis test). The homogeneous dispersion was checked among test groups (Supplementary Figure S3).

### Statistical Analysis and Data Visualization

Visualization was performed using R 4.0.3 software with the ggplot2 package. Categorical variables were described by the number of cases using the Chi-square test or Fisher’s exact test. If the continuous variables met normal distribution, they were described as mean ± standard deviation using independent samples *t*-test; if not, they were described as median (interquartile range) using the Mann Whitney U nonparametric test. Correlation analysis was performed using Spearman’s test. *p* < 0.05 was considered as statistically significant.

## Results

### Clinical Characteristics

Two groups of 40 patients, 20 in each group, were enrolled in our study, and their characterizations were showed in Supplementary Table 1. The male-to-female ratio is 4/16, and the age and gender of the two groups were matched.

Blood bilirubin in the bile reflux group increased compared to the control group (*p* < 0.05) but still normal. No clinical symptom, living habit or psychiatric factor were found to be significantly associated with bile reflux.

### Alterations of Oral Bile Acids Profile in REF Patients

**Figure 1:**
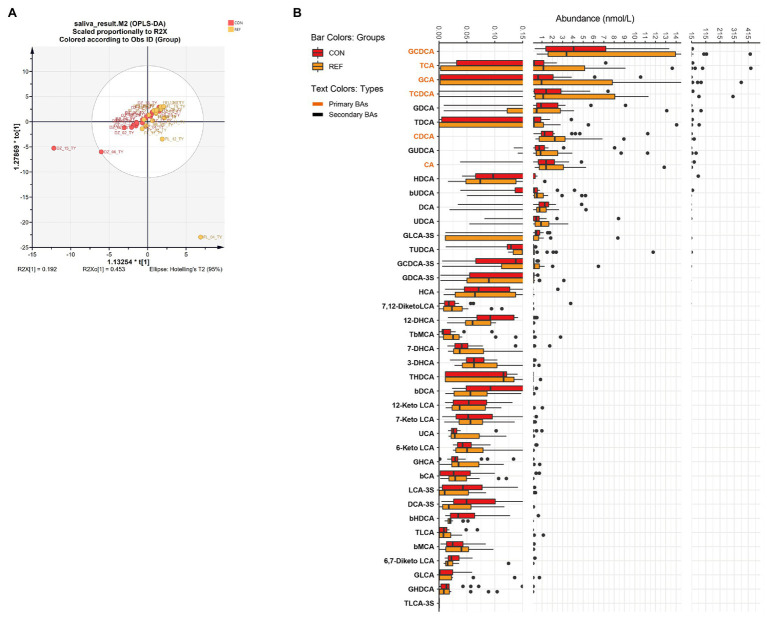
Alterations of oral bile acids profile. **(A)** Score plot of orthogonal projections to latent structures discrimination analysis (OPLS-DA). **(B)** Difference of bile acids levels. REF, bile reflux; CON, controls.

Orthogonal projections to latent structures discrimination analysis (OPLS-DA) were used to assess the bile acids alterations. There was no significant difference in the composition of salivary bile acids between the bile reflux group and the control group by OPLS-DA (Figure 1A; R2X[1] = 0.192, R2X_0_[1] = 0.453). Forty kinds of bile acids were detectable in saliva samples. Only four bile acid levels altered significantly in the REF group. The level of 12-DHCA, 6,7-diketo LCA, and β HDCA decreased while the level of TUDCA increased (Figure 1B).

### Increase in Gastric Bile Acids Under Bile Reflux

**Figure 2:**
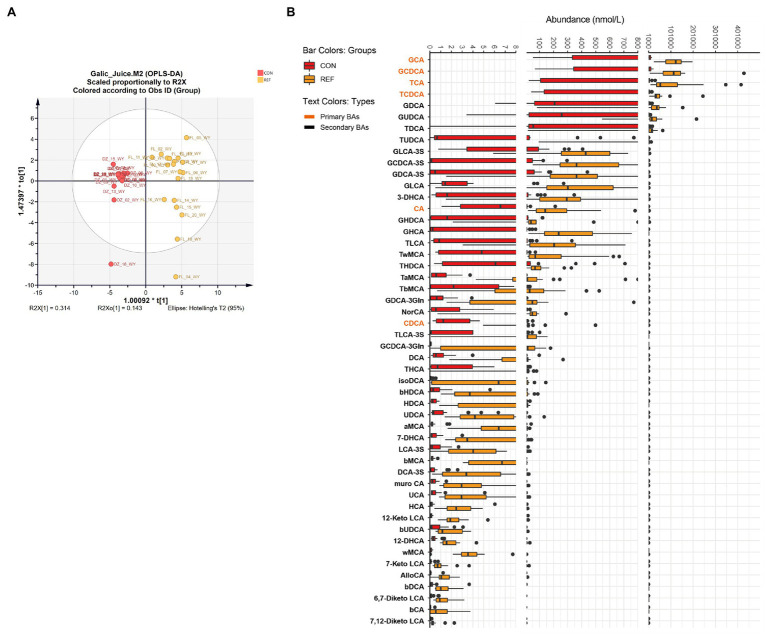
Alterations of gastric bile acids profile. **(A)** Score plot of orthogonal projections to latent structures discrimination analysis (OPLS-DA). **(B)** Difference of bile acids levels. REF, bile reflux; CON, controls.

OPLS-DA demonstrated that the bile acids composition of the gastric juice of bile reflux patients was significantly different from that of controls (Figure 2A; R2X[1]=0.314, R2X_0_[1]=0.143). Fifty kinds of bile acids were detected in gastric juice samples. Levels of primary bile acids and secondary bile acids were generally elevated in bile reflux patients. The changes of conjugated primary bile acids were most significant, such as βMCA, GCA, TCA, GHCA, GCDCA, TCDCA, and 7-DHCA (Figure 2B).

### Alterations of Fecal Bile Acids Profile in REF Patients

**Figure 3:**
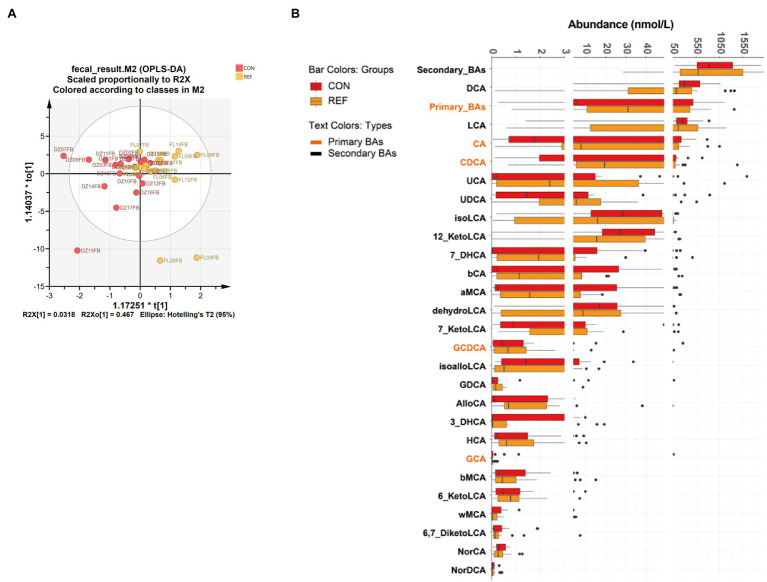
Alterations of fecal bile acids profile. **(A)** Score plot of orthogonal projections to latent structures discrimination analysis (OPLS-DA). **(B)** Difference of bile acids levels. REF, bile reflux; CON, controls.

OPLS-DA showed that fecal bile acid composition of bile reflux patients was not significantly different from the controls (Figure 3A; R2X[1]=0.318, R2X_0_[1]=0.467). Twenty-seven kinds of bile acids were detectable in all fecal samples, and no significant difference was detected in the fecal bile acids of bile reflux patients. AlloCA, 7-KetoLCA, 7-DHCA, CA, and CDCA increased, and iso-alloLCA decreased in bile reflux patients, but not statistically different (Figure 3B).

### The Spatial Profiling of Bacterial Community in the Gastrointestinal Tract

**Figure 4:**
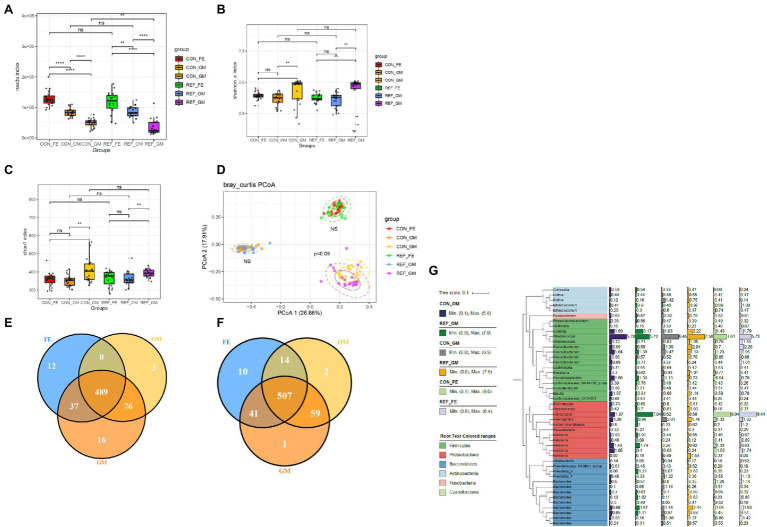
Alterations of bacterial microbiota profile in different part of digestive tract. **(A)** reads index. The alpha diversity, **(B)** Shannon index; **(C)** Chao1 index. ^*^p < 0.05. **(D)** Principal coordinate analysis (PCoA) of bacterial beta-diversity based on Bray Curtis distance. NS, not significant. The Venn diagram to visualize the common ASV in REF group **(E)** and CON group **(F)**. Phylogenetic tree **(G)**. REF, bile reflux; CON, controls; FE, feces; OM, oral mucosa; GM, gastric mucosa.

The reads measured in different parts of the digestive tract were not identical (Figure 4A). Feces had the highest reads index, followed by oral mucosa, whereas gastric mucosa contained the fewest bacteria.

Shannon index and chao1 index were used to assess the ɑ-diversity of the microbiota. Principal coordinate analysis (PCoA) was used for the β-diversity of the microbiota. The Shannon (Figure 4B) and chao1 indexes (Figure 4C) between different parts of the digestive tract were significantly different, as well as the composition pattern of the microbiota (Figure 4D). There were specific types of ASVs in different parts of the digestive tract. Oral mucosa and gastric mucosa contained more common ASVs, while oral mucosa and feces shared the least common ASVs (Figures 4E,F). We further analyzed the bacterial composition of the digestive tract in bile reflux and control groups, and found that Firmicutes, Proteobacteria, and Bacteroidetes were the main components at the phylum level (Figure 4G).

### Alterations of Oral Bacterial Microbiota Profile in REF Patients

**Figure 5:**
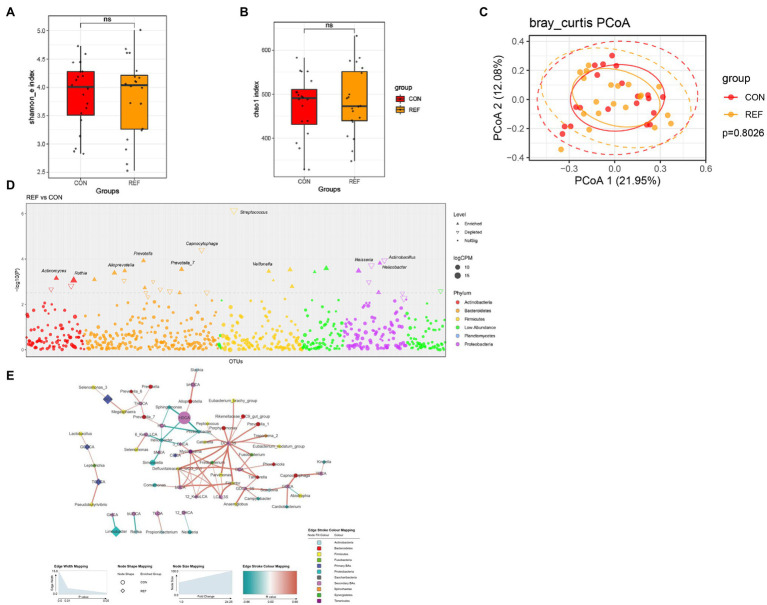
Alterations of oral bacterial microbiota profile. The alpha diversity, **(A)** Shannon index; **(B)** Chao1 index. ^*^p < 0.05. **(C)** PCoA of bacterial beta-diversity based on Bray Curtis distance. **(D)** Bacterial genera with abundance differentiation in REF group compared with CON group in the Manhattan diagram. Differences between two groups were shown as point shape indicated ASV enriched, depleted or not significant; point size indicated the abundance of ASV. **(E)** Co-occurrence network of bile acids and microbiota. The dot size indicated the enrichment degree in the REF group (diamond) and the CON group (circle). The thickness of the lines indicated the degree of positive (red) or negative (green) correlation. REF, bile reflux; CON, controls.

Shannon and chao1 indexes (Figures 5A,B) of oral mucosa microbiota between the REF group and the CON group were not statistically different. PCoA of beta diversity did not show separated clustering between the two groups (Figure 5C). The composition of microbiota of the REF and CON groups was analyzed in the Manhattan diagram at the genus level. The abundance of *Helicobacter*, *Prevotella*, and *Veillonella* remarkably increased. A significant reduction in the abundance of *Streptococcus*, *Capnocytophaga*, *Neisseria*, and *Actinobacillus* was observed in bile reflux patients compared with controls (Figure 5D).

To explore the correlation between bile acids and microbiota, we constructed a co-occurrence network based on Spearman’s correlation analysis. The results suggested correlations between the abundance of microbiota and bile acids in the oral cavity (Figure 5E).

### Stability of Gastric Bacterial Microbiota Under Bile Flux

**Figure 6:**
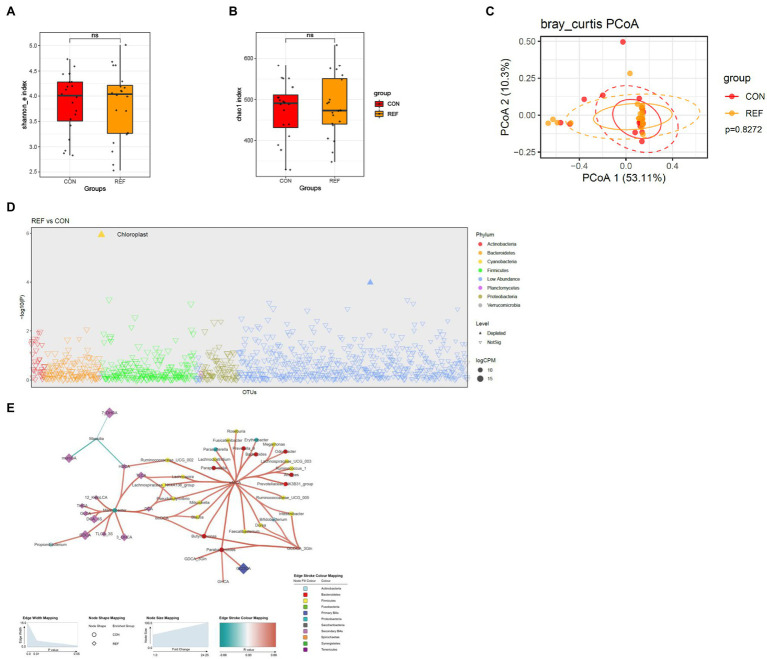
Alterations of gastric bacterial microbiota profile. The alpha diversity, **(A)** Shannon index; **(B)** Chao1 index. ^*^p < 0.05. **(C)** PCoA of bacterial beta-diversity based on Bray Curtis distance. **(D)** Bacterial genera with abundance differentiation in REF group compared with CON group in the Manhattan diagram. Differences between two groups were shown as point shape indicated ASV enriched, depleted or not significant; point size indicated the abundance of ASV. **(E)** Co-occurrence network of bile acids and microbiota. The dot size indicated the enrichment degree in the REF group (diamond) and the CON group (circle). The thickness of the lines indicated the degree of positive (red) or negative (green) correlation. REF, bile reflux; CON, controls.

The Shannon index (Figure 6A) and chao1 index (Figure 6B) of gastric mucosa microbiota between the REF and CON groups were not statistically different. PCoA of beta diversity also did not show much difference (Figure 6C). The gastric mucosal microbiota was stable, and only very few bacteria were altered in abundance under bile reflux compared to controls, namely an unassigned genus of the chloroplast (Figure 6D). There were also correlations between the abundance of microbiota and bile acids in the gastric cavity (Figure 6E).

Since Hp infection (Gantuya et al., 2019) significantly affected the composition of the gastric mucosal microbiota (Supplementary Figure S1), we excluded Hp-positive patients (four people in each group) and found that the results were similar as before (Supplementary Figure S2). Therefore, Hp infection did not interfere with the results.

### Alterations of Fecal Bacterial Microbiota Profile in REF Patients

**Figure 7:**
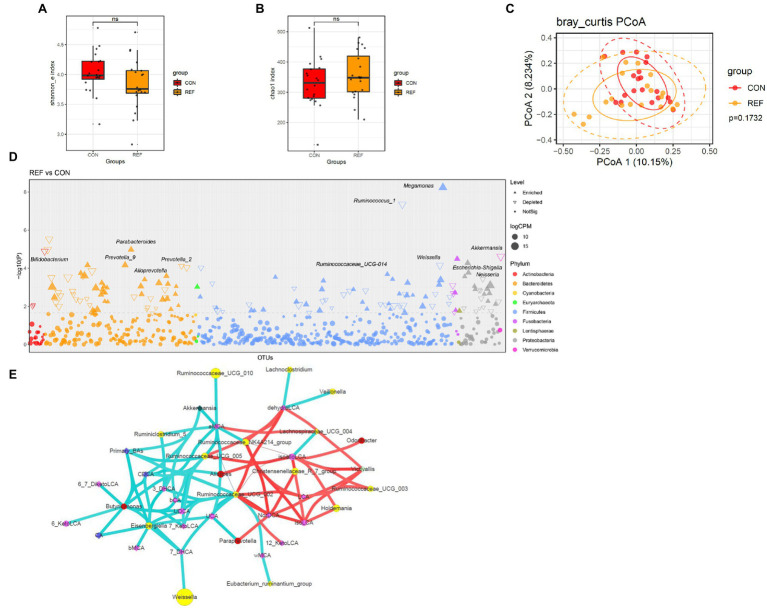
Alterations of fecal bacterial microbiota profile. The alpha diversity, **(A)** Shannon index; **(B)** Chao1 index. ^*^p < 0.05. **(C)** PCoA of bacterial beta-diversity based on Bray Curtis distance. **(D)** Bacterial genera with abundance differentiation in REF group compared with CON group in the Manhattan diagram. Differences between two groups were shown as point shape indicated ASV enriched, depleted or not significant; point size indicated the abundance of ASV. **(E)** Co-occurrence network of bile acids and microbiota. The dot size indicated the enrichment degree in the REF group (diamond) and the CON group (circle). The lines indicated the degree of positive (red) or negative (green) correlation. REF, bile reflux; CON, controls.

Shannon index (Figure 7A) and chao1 index (Figure 7B) of fecal microbiota between the REF group and the CON group were not significantly different. PCoA of beta diversity also did not show separated clustering between the two groups (Figure 7C). At the genus level, the abundance of *Bifidobacterium*, *Prevotella_2*, *Ruminococcus*, *Weissella*, *Neisseria*, and *Akkermansia* significantly decreased; while *Alloprevotella*, *Prevotella_9*, *Parabacteroides*, and *Megamonas* statistically increased (Figure 7D).

The correlations between microbiota and bile acids in feces were more complex than other parts of the digestive tract (Figure 7E). *Ruminococcaceae_UCG_002* was negatively correlated with primary bile acids and positively correlated with various secondary bile acids, which might convert primary bile acids into secondary bile acids.

## Discussion

Bile reflux patients commonly experience the retrograde movement of bile acids from the duodenum to stomach. To date, few studies have examined the bile acid profile of bile reflux patients along the length of the gastrointestinal tract or monitored segment specific changes to the microbiota. This study aimed to explore the characteristics of microbiota and bile acids profile in bile reflux patients.

OPLS-DA suggested little difference in the overall composition of salivary bile acids between bile reflux patients and controls, with only four bile acids altered. 12-DHCA, 6,7-diketoLCA, and β HDCA decreased, and TUDCA increased in bile reflux patients. TUDCA reduced endoplasmic reticulum stress by expanding the folding of proteins. In the oral cavity, TUDCA reduced the secretion of abnormal salivary mucins in Sjogren’s syndrome patients (Albornoz et al., 2017). There were little pieces of research on 12-DHCA and βHDCA. 6,7-diketoLCA decreased in end-stage renal disease patients and increased in gestational diabetes patients, but the function was unclear (Hou et al., 2018).

There were no significant differences in the α-diversity and β-diversity of oral bacteria between the bile reflux and control groups. *Streptococcus*, *Capnocytophaga*, *Neisseria*, and *Actinobacillus* significantly decreased in the oral mucosa of bile reflux patients. Approximately, 20% of the oral bacteria were *Streptococci*. *Streptococci* were part of the normal oral microbiota and commonly considered to be commensal organisms. *Streptococci* had the ability to metabolize a wide variety of carbohydrates and produce antimicrobial compounds, such as bacteriocins and hydrogen peroxide (Kreth et al., 2008; Wescombe et al., 2009). *Capnocytophaga* was commensal bacteria in the oral cavity of human and animals. A clinical research found that *Capnocytophaga* was present in 87% in healthy individuals, 77% in gingivitis and 73% in periodontitis. It seemed to be a conditional pathogen associated with gingivitis and periodontal disease (Pers and Frederiksen, 1996; Idate et al., 2020). The predominance of *Neisseria* indicated a healthy oral cavity condition (Zaura et al., 2009; Yamashita and Takeshita, 2017). The abundance of *Helicobacter*, *Prevotella*, and *Veillonella* significantly increased in the oral mucosa of bile reflux patients. Helicobacter pylori colonization in the oral cavity might be associated with the pathogenesis of halitosis, glossitis, and dental caries (Sayed et al., 2014). *Prevotella* mediated mucosal inflammation, leading to the systemic spread of inflammatory mediators and bacterial products (Larsen, 2017). *Veillonella* produced lipopolysaccharide (LPS), which activated the human complement system and led to the release of pro-inflammatory factors (Bui et al., 2019). Based on these results, we inferred that changes in the oral microbiota of bile reflux patients were characterized by an increased abundance of bacteria that promoted inflammation, whereas the abundance of commensal bacteria genera that predominated in the healthy oral cavity decreased. There were few studies on bile reflux and oral health. Whether the existence of the microbiota alteration contributed to oral symptoms or diseases was still unclear.

In accordance with previous studies (Zhao et al., 2020), gastric bile acid levels were generally higher in bile reflux patients than in controls, especially the conjugated primary bile acids.

There were no significant differences in the α-diversity and β-diversity of gastric bacteria between the bile reflux and control groups. Gastric mucosal microbiota was very stable under bile reflux. Considering that Hp infection might change the microbiota in the stomach, we excluded all Hp-positive patients and found similar results as before. In conclusion, bile reflux had little effect on the gastric mucosal microbiota, possibly due to the protective effect of the gastric mucus layer. The patients included in this study were primarily young and middle-aged adults, and gastroscopic pathology did not show a significant difference of inflammation or intestinal metaplasia in bile reflux patients, possibly because the duration of bile reflux was not long enough.

The fecal bile acids in bile reflux patients did not change statistically compared with controls. An increase in primary bile acids and a decrease in secondary bile acids were still found. Less fecal bile acids were detected than gastric juice and saliva, possibly due to DNA stabilizers in fecal collection tubes. Solvent might influence bile acids detection.

There were no significant differences in the α-diversity and β-diversity of fecal bacteria between the bile reflux group and the control group. *Bifidobacterium*, *Prevotella_2*, *Ruminococcus*, *Weissella*, *Neisseria*, and *Akkermansia* decreased significantly in fecal samples of bile reflux patients. *Bifidobacterium* was a commensal genus with various functions such as biological barrier, nutrition and immune regulation (Turroni et al., 2009). *Prevotella_2* was a minor genus in Prevotellaceae, associated with cardiovascular risk (Kelly et al., 2016), ankylosing spondylitis (Chen et al., 2019) and increased levels of C-reactive protein (Sun et al., 2019). *Ruminococcus gnavus* E1 in *Ruminococcus* produced a bacteriocin that inhibited the growth of pathogenic bacteria (Crost et al., 2011; Stefanie et al., 2019). *Weissella* made exopolysaccharides (EPS), reducing damage from oxidative stress (Bukola et al., 2018; Ishola and Adebayo-Tayo, 2018). *Akkermansia muciniphila*, a significant member of the *Akkermansia*, played a crucial role in promoting intestinal epithelial cell development (Kim et al., 2020). *Alloprevotella*, *Prevotella_9*, *Parabacteroides*, and *Megamonas* increased significantly in fecal samples of bile reflux patients. Previous studies found that *Alloprevotella* might be associated with intestinal inflammation and carcinogenesis (Wang et al., 2019). In a mouse model of colitis, *Prevotella_9* could lead to weight loss and intestinal epithelial inflammation (Hofer, 2014). *Parabacteroides* was able of bile acid conversion, converting conjugated bile acids to secondary bile acids and succinate (Wu et al., 2018). *Megamonas* increased in PSC patients with IBD and was associated with endoscopically active inflammation (Vaughn et al., 2019). Based on these results, the changes of fecal microbiota in REF patients were characterized by a decrease in commensal genera with anti-inflammatory and metabolically protective effects, as well as an increase in bacteria associated with inflammation and bile acid metabolism. However, bile reflux on the physiopathology of the intestine was not apparent, and further studies were needed.

We constructed a co-occurrence network based on Spearman’s correlation analysis. The results suggested that there was a correlation between microbiota and bile acids, especially in feces. *Ruminococcaceae_UCG_002* in feces was negatively correlated with primary bile acids and positively correlated with secondary bile acids, possibly related to the bile acids conversion. *Butyricimonas*, *Eisenbergiella*, and *Ruminococcaceae_UCG_002* were all enriched genera in the control group and were negatively correlated with primary bile acids. The decrease of primary bile acids in fecal samples of the control group might be associated with the enrichment of these genera. But there were no reports about the involvement of these genera in bile acid metabolism, and the mechanism of interaction with bile acids needed to be explored.

Bile reflux was not uncommon, but it did not receive enough attention and lacked uniform criteria for diagnosis and treatment. Our study suggested that bile reflux might negatively affect intestinal microecology, but whether the altered microbiota and bile acids in bile reflux patients could affect mucosal ecology or disease status was still unknown. More studies were still needed to explore the effects of bile reflux on humans and the mechanisms involved.

## Data Availability Statement

The 16s ribosomal RNA raw sequence data reported in this article have been deposited in the Genome Sequence Archive (Wang et al., 2017) of the National Genomics Data Center (National Genomics Data Center and Partners, 2020), Beijing Institute of Genomics (China National Center for Bioinformation), and Chinese Academy of Sciences under accession number CRA004485 and are publicly accessible at: https://bigd.big.ac.cn/gsa.

## Ethics Statement

The studies involving human participants were reviewed and approved by the Ethics Committee of Peking University People’s hospital. The patients/participants provided their written informed consent to participate in this study. Written informed consent was obtained from the individual(s) for the publication of any potentially identifiable images or data included in this article.

## Author Contributions

YL, NY, and JX designed this study. NY, YL, XW, NC, and LS performed the sample and clinical information collection. NY and JX performed the data analysis and visualization. NY performed the manuscript writing. YL and JX revised this article. All authors contributed to the article and approved the submitted version.

## Funding

This study was funded by the National Natural Science Foundation of China (nos. 82070539, 81873549 and 82000496), the Beijing Municipal Natural Science Foundation (no. 7214267).

## Conflict of Interest

The authors declare that the research was conducted in the absence of any commercial or financial relationships that could be construed as a potential conflict of interest.

## Publisher’s Note

All claims expressed in this article are solely those of the authors and do not necessarily represent those of their affiliated organizations, or those of the publisher, the editors and the reviewers. Any product that may be evaluated in this article, or claim that may be made by its manufacturer, is not guaranteed or endorsed by the publisher.

## Figures and Tables

**Table 1:** 

ALB	Albumin
ALT	Alanine aminotransferase
AST	Aspartate aminotransferase
ASV	Amplicon sequence variants
CA	Cholic acid
CDCA	Chenodeoxycholic acid
CHO	Cholesterol
DBIL	Direct bilirubin
DHCA	Dehydrocholic acid
DNA	Deoxyribonucleic acid
FBG	Fasting blood glucose
GAD-2	Generalized anxiety disorder-2
GCA	Glycocholic acid
GCDCA	Glycochenodeoxycholic acid
GHCA	Glycohyocholic acid
HBP	Hypertension
HDCA	Hyodeoxycholic acid
HLP	Hyperlipemia
IBD	Inflammatory bowel disease
IBIL	Indirect bilirubin
LCA	Lithocholic acid
LDL-C	Low density lipoprotein cholesterol
MCA	Muricholic acid
OPLS-DA	Orthogonal partial least squares-discriminant analysis
PCoA	Principal co-ordinates analysis
PHQ-2	Patient health questionnaire-2
PSC	Primary sclerosing cholangitis
RNA	Ribonucleic acid
TBIL	Total bilirubin
TCA	Taurocholic acid
TCDCA	Tauroursodeoxycholic acid
TG	Triglyceride
TUDCA	Tauroursodeoxycholic acid
T2DM	Type 2 diabetes mellitus
UA	Uric acid
UPLC-MS/MS	Ultra performance liquid chromatography tandem mass spectrometer
USA	United States of America
