# Supplementary material for: The Spatial Landscape of the Bacterial Community and Bile Acids in the Digestive Tract of Patients With Bile Reflux
Source: Front Microbiol. 2022 Mar 9;13:835310. doi: 10.3389/fmicb.2022.835310 (PMC8959417; doi:10.3389/fmicb.2022.835310)
Supplement: Supplementary file 1 [file Data_Sheet_1.PDF]

Supplementary Materials

Supplementary Table 1. Clinical characteristics of the patients

	Bile Reflux (n=20)	Control (n=20)	P value
Age	32.00(15.75)	35.50(14.25)	0.167
Gender(M:F)	4:16	4:16	1
BMI(kg/m^2)	21.32±3.40	22.57±3.57	0.265
**Complication**			
HBP	3(15%)	4(20%)	0.677
T2DM	0(0%)	1(5%)	0.311
HLP	2(10%)	2(10%)	1
Post-cholecystectomy	1(5%)	0	0.311
**Laboratory tests**			
ALT(U/L)	12.50(7.25)	16.50(12.5)	0.075
AST(U/L)	18.00(5.00)	20.00(6.00)	0.265
TBIL (umol/L) *	19.20±7.78	14.17±5.90	0.027
DBIL (umol/L) *	5.60(3.18)	4.20(1.97)	0.038
IBIL (umol/L) *	13.11±5.33	9.76±3.72	0.027
ALB(g/ L)	46.00±2.62	44.40±3.04	0.083
FBG(mmol/L)	5.14(0.26)	5.09(0.44)	0.862
CHO(mmol/L)	4.78±1.03	4.95±0.82	0.576
TG(mmol/L)	0.83(0.45)	1.25(0.71)	0.600
LDL-C(mmol/L)	2.91±0.89	3.05±0.59	0.539
UA(umol/L)	308.35±51.48	309.25±86.05	0.968
**Symptoms**			
Nause	0.00(1.00)	0.00(0.75)	0.529
Vomitting	0.00(0.00)	0.00(0.00)	0.583
Bloating	0.50(1.00)	1.00(1.00)	0.883
Abdominal pain	1.00(1.00)	0.00(1.00)	0.678
Early satiety	0.00(1.00)	0.00(1.00)	0.547
Heartburn	0.00(1.00)	1.00(1.00)	0.883
Loss of appetite	0.00(1.00)	0.00(0.00)	0.231
Bitterness	0.00(1.00)	0.00(0.75)	0.512
**Psychological score**			
PHQ-2	1.00(1.75)	1.50(2.00)	0.862
GAD-2	1.00(3.00)	1.00(2.00)	0.445
**Living habbits**			
Breakfast (day/w)	7.00(1.50)	7.00(0.00)	0.779
Midnight eating(day/w)	0.00(0.00)	0.00(0.00)	0.779
Regular eating(day/w)	7.00(1.50)	7.00(2.00)	0.841
Coffee (time/w)	0.00(3.75)	0.00(0.75)	0.192
Soda(time/w)	0.00(2.00)	0.00(1.00)	0.779

Spicy food(time/w)	2.00(3.00)	1.00(1.00)	0.968
Greasy food (time/w)	1.50(3.00)	1.50(2.00)	0.841
Exercise (time/w)	0.00(1.75)	0.50(2.00)	0.547
Sleeping hour(h)	7.00(0.88)	7.25(1.00)	0.815
Stay up late	14(70%)	9(45%)	0.11
Smoking	4(20%)	2(10%)	0.376
Alcohol consumption	6(30%)	4(20%)	0.465
**Pathology/Hp infection**			
Inflammation	1.00(0.75)	1.00(0.00)	0.779
Intestinal metaplasia	0.00(0.00)	0.00(0.00)	0.799
Atrophy	0.00(0.00)	0.00(0.00)	0.583
Hp infection	4(20%)	4(20%)	1

Patients were asked by the doctor to rate the intensity of each individual symptom on a validated 5-point Likert scale (0 = no problem, 1 = mild problem, 2 = moderate problem, 3 = severe problem and 4 = very severe problem). PHQ-2 asked about the frequency of the symptoms of depressed mood and anhedonia, scoring 0–3 (“not at all [0]”, “several days [1]”, “more than half the days [2]”, “nearly every day [3]”); GAD-2 asked about the frequency of the symptoms of anxious mood and uncontrollable worrying, scoring 0–3 (“not at all [0]”, “several days [1]”, “more than half the days [2]”, “nearly every day [3]”). Pathological grading was based on the updated Sydney System. Hp infection was determined by positive rapid urease test or positive pathological staining.

Supplementary Table 2. The process of bacterial sequence analysis

	ALL	Oral Mucosa	Gastric Mucosa	Feces
**Quality control**				
Kept sequences	11641890	3669663	2523798	5448429
discarded Sequences	1031856	362706	141711	527439
**Dereplication**				
cutoff	120	24	24	30
nt	4721152083	1538564813	947193792	2235393478
seqs	11641890	3669663	2523798	5448429
min	210	210	210	210
max	450	450	450	450
avg	406	419	375	410
unique sequences	4895606	1513412	1101469	2382339
uniques written	6606	9058	6573	8930
cluster discarded	4889000	1504354	1101469	2373409
**ESV denoise**				
size	11Mb	22Mb	11Mb	22Mb
total amplicons	638	668	724	664
good amplicons	638	653	723	632
chimeras	0	15	2	32
**Chimeras removal**				
db	silva_16s_v123	silva_16s_v123	silva_16s_v123	silva_16s_v123
amplicons	638	653	723	632
chimeras	3(0.5%)	4(0.6%)	140(21.4%)	4(0.6%)
in db	529(82.8%)	509(77.9%)	323(44.7%)	526(83.2%)
not matched	106 (16.6%)	140(21.4%)	397 (54.9%)	102(16.1)
**ASVtab generation**				
nt	257019	269744	256425	258370
seqs	635	649	720	628
min	210	247	210	383
max	445	427	450	427
avg	405	416	356	411
unique sequences	11641890	3669663	2523798	5448429
query sequence	9955973(85.52%)	3495977(95.27%)	1838681(72.85%)	5031930(92.36%)
**ASVtab stats**				
reads	10740175	3495977	1838681	5031930
samples	120	40	40	40
ASVs	4295	649	720	628
count	515400	25960	28800	25120
count=0	393509	8136	4217	11959
count=1	24552	1718	1071	2317
count>=10	47444	11532	16542	7664
ASVs found in all samples	4(0.1%)	66(10.2%)	222(30.8%)	40(6.4%)
ASVs found in 90% of samples	26(0.6%)	200(30.8%)	445(61.8%)	116(18.5%)

ASVs found in 50% of samples	322(7.5%)	502(77.3%)	673(93.5%)	326(51.9%)
**sample size**				
min	16826	56480	14292	48021
low	64290	75762	28836	108324
med	85794	84275	50771	126987
mean	89501.5	87399.4	45967	125798.3
hi	116738	99414	59197	144822
max	210831	128709	127550	208306
**taxonomy**				
Phylum	13	12	11	9
Class	21	19	18	16
Order	32	34	34	21
Family	52	52	60	37
Genus	128	81	121	112
