# Supplementary material for: The Spatial Landscape of the Bacterial Community and Bile Acids in the Digestive Tract of Patients With Bile Reflux
Source: Front Microbiol. 2022 Mar 9;13:835310. doi: 10.3389/fmicb.2022.835310 (PMC8959417; doi:10.3389/fmicb.2022.835310)

Supplementary Figure S1. Alterations of bacterial microbiota profile in Hp-positive patients. (A) reads index. The alpha diversity, (B) Shannon index; (C) Chao1 index. *p < 0.05. (D) Principal coordinate analysis (PCoA) of bacterial beta-diversity based on Bray Curtis distance. POS, Hp-positive; NEG, Hp-negative; FE, feces; OM, oral mucosa; GM, gastric mucosa.

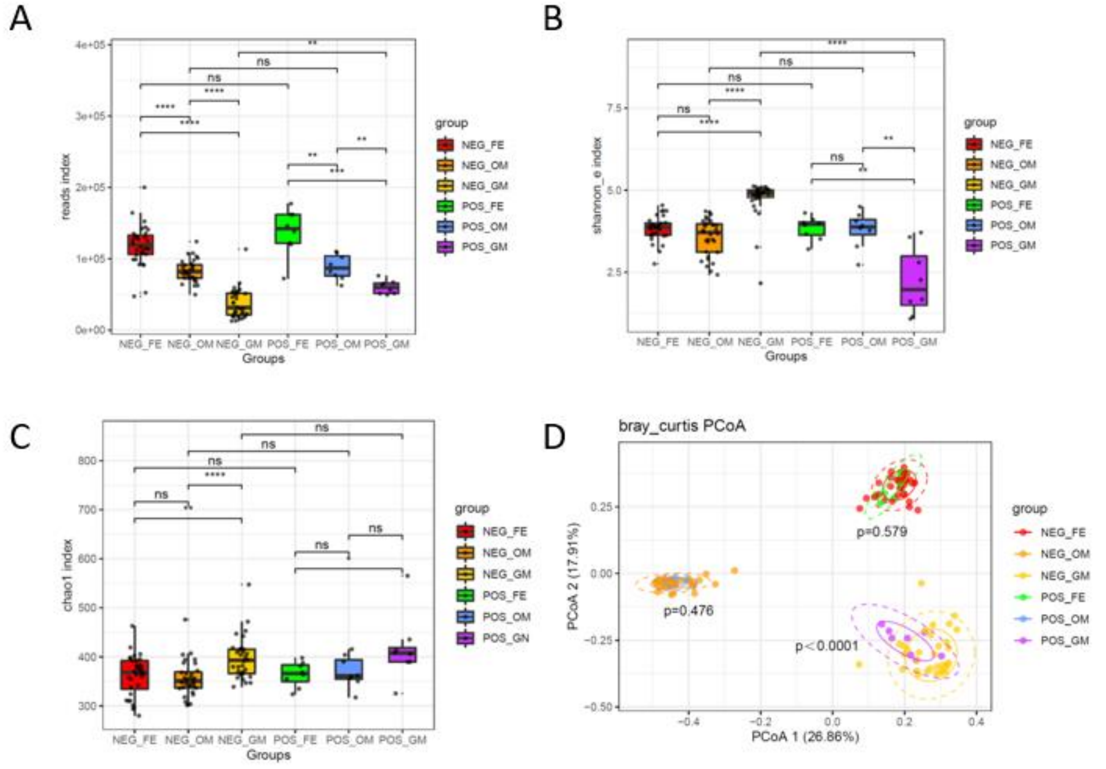

Supplementary Figure S2. Alterations of gastric bacterial microbiota profile under bile reflux in Hp-positive and Hp-negative patients. The alpha diversity, (A) Shannon index; (B) Chao1 index. *$p < 0.05$. (C) PCoA of bacterial beta-diversity based on Bray Curtis distance. (D)Bacterial genera with abundance differentiation in REF group compared with CON group of Hp-negative individuals. Differences between two groups were shown as point shape indicated ASV enriched, depleted or not significant; point size indicated the abundance of ASV. REF, bile reflux; CON, controls; POS, Hp-positive; NEG, Hp-negative.

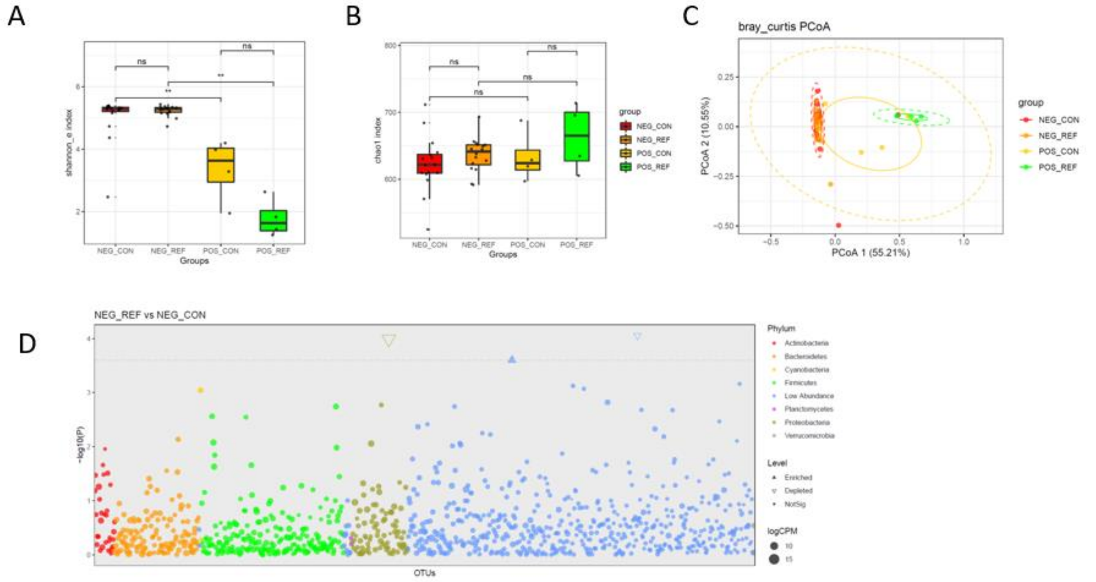

Supplementary Figure S3. Group multivariate dispersions of beta diversity. (A) Oral mucosa; (B) Gastric mucosa; (C)Feces.

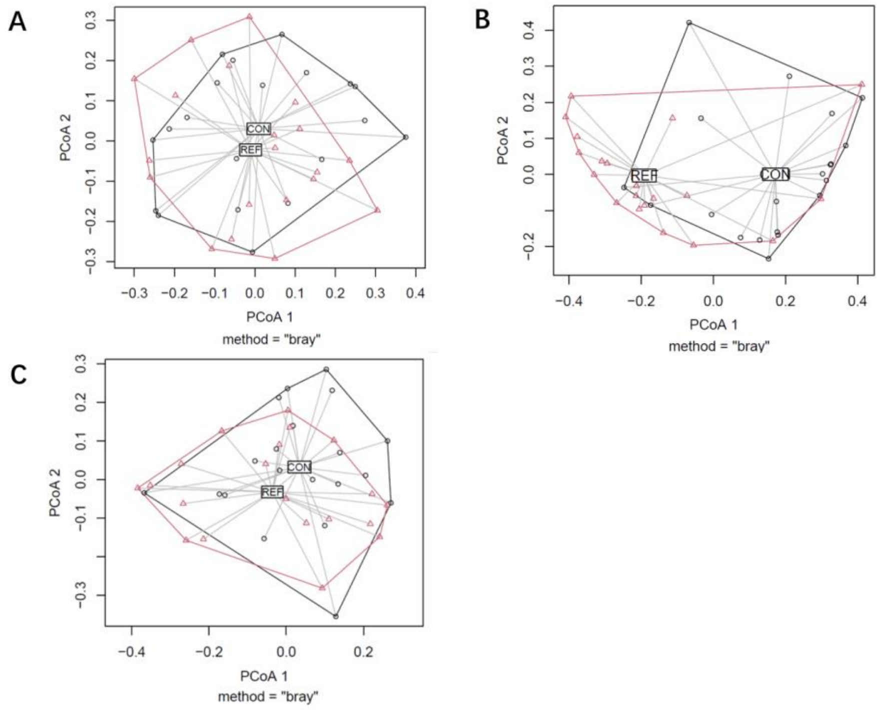

Supplement: Supplementary file 2 [file Data_Sheet_2.PDF]
